# Hypertension and overall survival in metastatic colorectal cancer patients treated with bevacizumab-containing chemotherapy

**DOI:** 10.1038/bjc.2011.2

**Published:** 2011-02-08

**Authors:** P Österlund, L-M Soveri, H Isoniemi, T Poussa, T Alanko, P Bono

**Affiliations:** 1 Department of Oncology, Helsinki University Central Hospital, P.O. Box 180, HUS Helsinki 00029, Finland; 2 Department of Surgery, Helsinki University Central Hospital, Helsinki, Finland; 3 STAT-consulting, Nokia, Finland; 4 Laboratory of Molecular Oncology, Molecular Cancer Biology Research Program, University of Helsinki, Helsinki, Finland

**Keywords:** bevacizumab, hypertension, predictive factor

## Abstract

**Background:**

Hypertension (HTN) is a common toxicity of anti-VEGF (vascular endothelial growth factor) antibody treatment. It may be a marker of VEGF signalling pathway inhibition and therefore represent a cancer biomarker in metastatic colorectal cancer (mCRC) patients treated with chemotherapy and bevacizumab.

**Methods:**

A total of 101 consecutive patients with mCRC were treated with standard chemotherapy combined with bevacizumab at dose of 2.5 mg kg^−1^ per week in a single centre. The median follow-up time of the patients alive was 64 months. Blood pressure was measured before each bevacizumab infusion, and HTN was graded according to common toxicity criteria for adverse events version 3.0.

**Results:**

Overall, 57 patients (56%) developed ⩾grade 1 HTN (median blood pressure 168/97 mm Hg), whereas 44 (44%) remained normotensive when treated with bevacizumab-containing chemotherapy regimen. Overall response rate was higher among patients with HTN (30 *vs* 20% *P*=0.025). Hypertension was associated with improved progression-free survival (10.5 *vs* 5.3 months; *P*=0.008) and overall survival (25.8 *vs* 11.7 months; *P*<0.001), and development of HTN within 3 months had an independent, prognostic influence in a multivariate landmark survival analysis together with other known mCRC prognostic factors (*P*=0.007). There was no association between HTN and development of thromboembolic complications.

**Conclusion:**

Hypertension may predict outcome of bevacizumab-containing chemotherapy in mCRC. These data require confirmation in prospective studies including pharmacodynamic and pharmacokinetic analyses.

Bevacizumab is an anti-VEGF (vascular endothelial growth factor) monoclonal antibody shown to be effective in the treatment of metastatic colorectal cancer (mCRC). According to several randomised studies bevacizumab prolongs both progression-free and overall survival (OS) (Ranpura *et al*, 2010). In all studies, hypertension (HTN) has been recognised as a common adverse event associated with bevacizumab treatment (Ranpura *et al*, 2010).

Although not all patients diagnosed with mCRC do benefit from anti-VEGF antibody treatment, there are currently no biomarkers available for the prediction of the efficacy of antiangiogenic therapies. Hypothetically, HTN might be such a predictor, as VEGF signalling pathway inhibition removes endothelial cell survival signal leading to apoptosis and capillary rarefaction. It also reduces the amount of endothelial cell-derived nitric oxide, causing vascular smooth muscle constriction, increased vascular resistance, and elevated blood pressure (Dvorak, 2002). Recently, bevacizumab-associated HTN has been suggested to predict treatment efficacy in metastatic renal cell cancer patients treated with single-agent bevacizumab or sunitinib (Bono *et al*, 2009; Rini *et al*, 2010b). In this study, we investigated whether treatment-related HTN is associated with outcome and safety in 101 consecutive mCRC patients treated with bevacizumab-containing chemotherapy in a single centre.

## Patients and methods

### Patients and treatment

Bevacizumab has been used as a standard treatment in mCRC at the Department of Oncology, Helsinki University Hospital (HUCH), since April 2004. During the inclusion period (April 2004–December 2005), 114 consecutive patients treated with chemotherapy and bevacizumab were recognised. No patients were excluded due to baseline HTN. Hypertension and response data during at least 2 cycles of bevacizumab-containing therapy were available in 101 of these patients. Eleven patients were excluded due to short treatment duration (0.07–1.08 months). Reasons for early discontinuation were clinical progression without radiological assessment and sufficient blood pressure measurements (*n*=5) or toxicity leading to early cessation of therapy (venous thromboembolism, *n*=2; myocardial ischaemia, *n*=2; bowel perforation, *n*=1; proteinuria, *n*=1). Two patients had non-evaluable disease.

At the time of inclusion, the following criteria were used as contraindication for bevacizumab treatment: brain metastases, high-dose NSAIDs, prior pulmonary embolism or recent venous thromboembolic event, any arterial thromboembolic event, or baseline ⩾grade 2 proteinuria. Data cut-off was set at 24 November 2010, and by that time 91 (90%) patients had died. The median follow-up time for patients alive (*n*=10) was 63.5 months (range 20.6–73.6 months). This retrospective, single-centre exploratory study was approved by an Institutional Review Board.

The dose of bevacizumab was 5 mg kg^−1^ every 2 weeks (*n*=72) or 7.5 mg kg^−1^ every 3 weeks (*n*=29). None of the patients had received prior antiangiogenic therapy. Sixty patients (59%) received bevacizumab with 5-FU/LV/irinotecan (FOLFIRI) and the rest of the patients received bevacizumab combined with single-agent irinotecan or irinotecan+capecitabine (*n*=21), oxaliplatin-based therapy (*n*=12), or 5-FU-based therapy (*n*=7). One dihydropyrimidine dehydrogenase-deficient patient received single-agent bevacizumab.

Seventy eight (77%) of the patients received further systemic therapy after progression on bevacizumab-containing regimen. All of the three most important chemotherapeutic agents for mCRC (5-FU, oxaliplatin, and irinotecan) were used at some phase of the treatment in 86 (85%) of the patients, in 88% of the hypertensive and 82% of the normotensive patients (*P*=0.41). Bevacizumab beyond progression was given to 14 (14%) patients. Only 17 patients (17%) received cetuximab treatment after bevacizumab failure.

### Assessment of efficacy, side effects, and blood pressure

Patient demographics, adverse events, hospitalisations, and treatment efficacy were available and collected from all patients. Treatment response was evaluated according to RECIST criteria by whole body spiral computerised tomography performed every 8–10 weeks.

Blood pressure was measured by a nurse in resting position (after minimum 10 min rest) at baseline and before each bevacizumab infusion (starting at 14–21 days from treatment initiation) in an outpatient department. If HTN emerged in a single measurement, patients measured blood pressure 2–4 times weekly at home. Blood pressure data was filed at baseline and every 3 months, thereafter until 2 years. Three monthly was chosen because a new cycle is due at this time point in both 2 and 3 weekly dosing. Early HTN was additionally recorded at 1 month; that is, at 3 or 4 weeks depending on the schedule.

Hypertension was defined according to the common toxicity criteria for adverse events version 3.0 (CTCAE v3.0) as follows: Grade 1, transient (<24 h), asymptomatic, blood pressure rise by 20 mm Hg (diastolic) or to >150/100 mm Hg, if previously normal; Grade 2, recurrent, persistent, or symptomatic rise in diastolic blood pressure >20 mm Hg or systolic blood pressure rise to >150/100 mm Hg, if previously normal (monotherapy with antihypertensive agents may be indicated); Grade 3, requiring more than one drug therapy or more intensive therapy than previously.

### Statistical analyses

The variables with non-normal distributions were compared with the Mann–Whitney *U*-test, and the *χ*^2^-test was used for categorical variables.

The association between HTN and OS and progression-free survival (PFS) was described using the Kaplan–Meier survival curves, and the log-rank test was used for group comparisons. Overall survival was defined as the time from the date of initiation of bevacizumab-containing therapy to the date of death from any cause. Progression-free survival was defined as the time from the date of initiation of the bevacizumab-containing therapy to the day of documented disease progression or death due to any cause. Patients without documented event were censored at last follow-up, and two patients were censored at time of metastasectomy with curative intent.

The landmark survival analysis (with the landmark set at 3 months after date of initiation of bevacizumab-containing therapy) was performed. The landmark method was applied to avoid the bias caused by the time-dependent definition of HTN. Overall survival time was defined as the time from the landmark time to the date of death from any cause. For survival times, two patients were censored at the time of operation with curative intent, and one patient was excluded due to death before the landmark time point.

The univariate analyses were performed using the Cox proportional hazards models with HTN status at the landmark time and baseline characteristics (such as age, gender, primary tumour, line of treatment, baseline WHO performance status, number of metastatic sites) as independent variables. Then, the statistically significant (*P*<0.05) prognostic variables given by the univariate analyses were included as covariates together with HTN status in the multivariate Cox model. The results are given as hazard ratios (HR) with 95% confidence intervals (CI_95%_).

All statistical analyses were performed using a StatView statistical package (Berkley, CA, USA), sigma-plot version 11 (Systat software corporation, Chicago, IL, USA), or SPSS version 18.0 (SPSS Inc., Chicago, IL, USA).

## Results

### Patient population and efficacy of the treatment

In total, 101 patients, who initiated bevacizumab-containing treatment for mCRC between April 2004 and December 2005, were evaluated. Patient baseline demographics are shown in Table 1.

The median duration of bevacizumab-containing therapy was 8.6 months (range, 1.2–70.5). For patients who received bevacizumab as part of the first-line treatment (*n*=33), the median duration of therapy was 12.0 months (range, 1.2–68) compared with 7.5 (range, 1.3–70.5) and 6.6 months (range, 1.9–27) for those who received bevacizumab as second (*n*=39) or later; that is, third- to seventh-line (*n*=29) therapy. At the time of analysis (median follow-up 63.5 months), 100 (99%) of the patients had progressed and 90 (89%) had died. There were no treatment-related deaths.

The overall response rate (RR) was 50% and the disease control rate 89%. Radiological complete response was achieved in six (6%) patients. The median PFS was 8.8 months (CI_95%_: 8.0–9.6) and OS 18.9 months (CI_95%_: 15.1–22.7).

### Effect of HTN on outcome

A total of 57 (56%) patients were diagnosed with ⩾grade 1 HTN (grade 1 (*n*=26), grade 2 (*n*=28), and grade 3 (*n*=3)), whereas the rest of the patients stayed normotensive (grade 0 (*n*=44)) during bevacizumab treatment. Forty two (74%) of these ⩾grade 1 HTN patients were normotensive at baseline and 15 (26%) were normotensive with antihypertensives at baseline, but HTN recurred during bevacizumab-containing treatment.

General guidelines for treatment of HTN were implemented and antihypertensives were used in 30 patients. No group of antihypertensives was favoured. Diuretics (*n*=9), ACE/AT blockers (*n*=12), Ca-blockers (*n*=6) and *β*-blockers (*n*=12), and other antihypertensives (*n*=2) were used alone or in combination (*n*=9) making comparisons between drugs unmeaningful. Medication was prescribed to six antihypertensives-naive patients and intensified in one.

The frequency of the most important prognostic factors (including treatment line, age, and WHO performance status) were similar between hypertensive and normotensive patients. However, the number of metastatic sites differed between the groups (Table 1).

**Figure 1:**
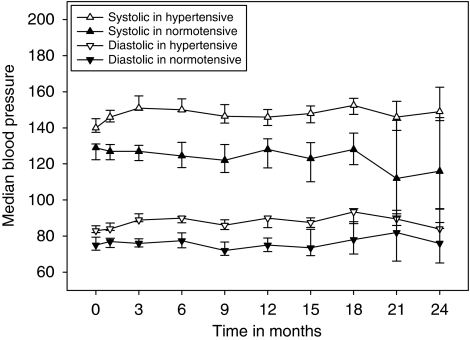
Systolic and diastolic blood pressure during bevacizumab treatment in hypertensive (*n*=57) and normotensive (*n*=44) patients. Median and 95% confidence interval.

The median time to the onset of HTN was 1 month (range, 1–15 and within 6 months in 95%) as calculated from the start of bevacizumab treatment. The median values of systolic and diastolic blood pressure before and during bevacizumab-containing treatment are presented in Figure 1. There were no statistically significant differences in the frequency of ⩾grade 3 adverse events, non-haematologic (21 *vs* 20, *P*=0.90), and haematologic (20 *vs* 14, *P*=0.56), or arterial thromboembolic events (0 *vs* 2, *P*=0.10), between patients with (*n*=57) or without HTN (*n*=44).

**Figure 2:**
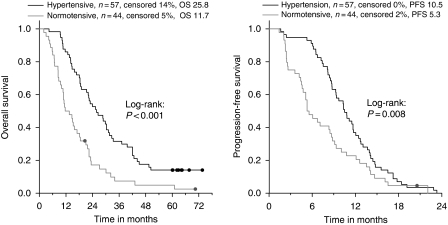
Overall survival and progression-free survival by hypertension during bevacizumab-containing treatment.

Effect of HTN on patient outcomes (RR, PFS, and OS) are presented in Table 2. Patients (*n*=57) who developed ⩾grade 1 HTN, had significantly longer OS than patients with no HTN during the treatment (*P*<0.001); the median OS was 25.8 months (CI_95%_: 19.2–32.4) for the patients with HTN *vs* 11.7 months (CI_95%_: 7.2–16.1) for the normotensive (Figure 2). A similar difference was also detected when PFS and OS of patients with grade 0–1 HTN were compared with OS and PFS of patients with grades 2–3 HTN.

**Figure 3:**
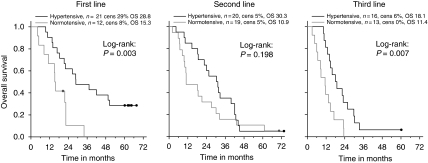
Effect of hypertension on overall survival by line of treatment.

A similar prognostic influence of HTN on OS was detected, regardless of the treatment line as shown in Figure 3. Median OS of first-line patients with treatment-related HTN was 28.8 (CI_95%_: 12.8–44.8) and 15.3 (CI_95%_: 14.0–16.6) months among patients with no HTN (*P*=0.291). In second-line bevacizumab treatment, the OS was 30.3 (CI_95%_: 19.8–40.9) months among patients with HTN and 10.9 (CI_95%_: 0.36–21.5; *P*=0.028) among patients with no HTN, whereas in third- to seventh-line treatment the OS was 18.1 (CI_95%_: 12.1–24.0) *vs* 11.4 months (CI_95%_: 7.5–15.3; *P*=0.007), respectively.

A PFS benefit was also noted in favour of the hypertensive group, 10.5 (CI_95%_: 9.0–12.0) *vs* 5.3 months (CI_95%_: 3.5–7.1; *P*=0.008; Figure 2). Furthermore, HTN was associated with significantly more responses (30 *vs* 20% *P*=0.025). In patients with ⩾grade 1 HTN, only 4% had PD as best response, whereas 20% of the non-HTN patients had PD as best response.

### Early HTN during bevacizumab therapy is predictive for survival

**Figure 4:**
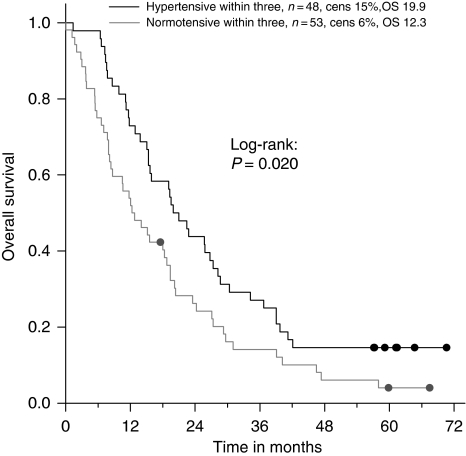
The landmark method determines each patient's HTN status at 3 months (HTN, *n*=48; no HTN, *n*=53). Overall survival time is calculated from that time point.

We also performed univariate and multivariate analysis to find out whether HTN within 3 months was an independent predictive factor for treatment efficacy. We used the landmark model in which each patient's HTN status at 3 months (HTN, *n*=48 and no HTN, *n*=53) was determined. Survival was calculated from that time point. One patient who had died before this landmark time point was excluded from further analyses. Median OS for patients with HTN at 3 months was 19.9 (CI_95%_: 15.7–24.1) and in no HTN patients 12.3 months (CI_95%_: 6.9–17.7; *P*=0.020; Figure 4).

In univariate analysis, HTN within 3 months (HR 1.73, *P*=0.011) was a predictive factor for OS as well as WHO performance status, line of treatment, number of metastatic sites, and type of chemotherapy (Table 3). These significant factors were added in a multivariate analysis, and bevacizumab treatment-related HTN was an independent predictive factor (HR 0.53; *P*=0.007). Other predictive factors were line of treatment and number of metastatic sites, whereas WHO performance status and type of chemotherapy were no longer predictive (Table 3).

## Discussion

Bevacizumab-containing chemotherapy has been considered as a standard treatment for mCRC since Hurwitz *et al* (2004) published the pivotal study of the efficacy and tolerability of bevacizumab in combination with irinotecan and 5-FU. However, not all patients benefit from bevacizumab, but at present there are no tools to predict the benefit of the addition of bevacizumab to chemotherapy in mCRC. Different biomarkers such as B-raf and K-ras mutation, microvessel density, VEGF, or VEGFR expression have been widely studied, but so far no predictive factors have been identified (Ince *et al*, 2005; Jubb *et al*, 2006).

Most common adverse events related to bevacizumab include proteinuria, thromboembolic and wound healing complications, gastrointestinal perforations and HTN. Data from pooled randomised trials have shown that combination treatment with bevacizumab and chemotherapy, compared with chemotherapy alone, is associated with an increased risk of arterial thromboembolism (Scappaticci *et al*, 2007). Although HTN has traditionally been considered as a side effect of the treatment, it may represent a measure of efficacy rather than toxicity. This assumption is supported by our results as well as observations that mean systolic and diastolic blood pressure of patients receiving VEGF signalling pathway inhibitors increase with exposure to these drugs and return to baseline after cessation of the treatment (Maitland *et al*, 2009). Studies by Maitland *et al* (2009) suggest that the wide variability in blood pressure responses to VEGF pathway inhibitors reflect pharmacodynamic variability. Additional data are needed to address the exact mechanisms related to HTN in these patients and to address whether there is a relationship between HTN induced by various VEGF pathway inhibitors and the incidence of cardiovascular side effects including reduced left ventricular ejection fraction.

According to a recently published large meta-analysis (Ranpura *et al*, 2010), the incidence of HTN in bevacizumab-treated cancer patients was 23.6 with 7.9% being grade 3–4. Bevacizumab treatment-related HTN is a very interesting subject at the moment. We have earlier reported that HTN during bevacizumab treatment in metastatic renal cell carcinoma (RCC) is associated with better outcome. Similar data in metastatic RCC, breast cancer, and lung cancer have also been published by others very recently (Schneider *et al*, 2008; Dahlberg *et al*, 2010; Rini *et al*, 2010a). In patients with mCRC, according to our knowledge, treatment-related HTN is a subject very rarely evaluated even though bevacizumab is a routine treatment in mCRC and in a wide use. Scartozzi *et al* (2009) have conducted a retrospective study in a small patient population (*n*=39), in which eight patients developed HTN and were analysed in detail. Our study identified 57 patients with HTN (56% of the patients) and enabled us to do a considerably larger comparison between patients with HTN and no HTN during bevacizumab-containing treatment.

We have shown that early HTN, within 3 months of treatment initiation, is predictive for OS. Similarly, Dahlberg *et al* (2010) have shown that HTN within 1 month in bevacizumab therapy for lung cancer is predictive for survival. Thus, it might be justifiable to reconsider the continuation of bevacizumab at the first response evaluation, and cessation in the absence of HTN may be wise if the patient has poor tolerability, if economical restraints for the use of the drug exist, or if alternative therapies are available, for example, EGFR inhibitors.

It would have been interesting to monitor the blood pressure more thoroughly also in this study, but the procedure used, measuring blood pressure once before each infusion, was as recommended in the Summary of Product Characteristics. Prospective studies including ambulatory blood pressure monitoring and pharmacodynamic analyses are also required in mCRC. These could be able to disclose whether blood pressure elevation predicts outcome and what is the source of pharmacodynamic variability. As certain VEGF genotypes may protect against VEGF signalling pathway inhibitor-induced HTN (Schneider *et al*, 2008), such prospective studies may also find new strategies for identifying patients at-risk for cardiovascular toxicities.

In conclusion, our results show that HTN predicted bevacizumab treatment efficacy regardless of the analysed end point (OS, PFS, or RR). It should be remembered that HTN is a known risk factor for cardiovascular complications and therefore effective treatment of bevacizumab-related HTN is recommended. (Ranpura *et al*, 2010).

## Figures and Tables

**Table 1:** Patient baseline demographics

	**All, *n*=101 (%)**	**No hypertension, *n*=44 (44%)**	**Hypertension grade 1–3, *n*=57 (56%)**	***P*-value**
*Median age, years (range)*	59.0 (35–79)	56.4 (35–76)	61.0 (43–79)	0.051^a^
*Gender*				0.162^a^
Male	54 (53)	27 (61)	27 (47)	
Female	47 (47)	17 (39)	30 (53)	
*Primary site*				0.517^a^
Colon	56 (55)	26 (59)	30 (53)	
Rectal	45 (45)	18 (41)	27 (47)	
*WHO performance status*				0.192^a^
0	33 (33)	11 (25)	22 (39)	
1	57 (56)	26 (59)	31 (54)	
2	11 (11)	7 (16)	4 (7)	
*No. of metastatic sites*				0.0020^a^
1	33 (33)	7 (16)	26 (46)	
2	17 (17)	9 (20)	8 (14)	
⩾3	51 (50)	28 (64)	23 (40)	
*Line of treatment*				0.567^a^
First	33 (32)	12 (27)	21 (37)	
Second	39 (39)	19 (43)	20 (35)	
Third–seventh	29 (29)	13 (30)	16 (28)	
*Chemotherapy used*				0.321^a^
5-Fluorouracil based	7 (7)	5 (11)	2 (3)	
Irinotecan based	81 (80)	35 (80)	46 (81)	
Oxaliplatin based	12 (12)	4 (9)	8 (14)	
No chemotherapy	1 (1)	0 (0)	1 (2)	

a *χ*^2^-test.

**Table 2:** Treatment efficacy in the overall study population and in subgroups divided by hypertension

	**All (*n*=101)**	**No hypertension, *n*=44 (44%)**	**Hypertension grade 1–3, *n*=57 (56%)**	***P*-value**
*Response rate* ^*^				0.025^a^
CR/PR	50	20	30	
SD	40	15	25	
PD	11	9	2	
Progression-free survival (months)	8.8	5.3	10.5	0.008^b^
Overall survival (months)	18.9	11.7	25.8	<0.001^b^

a *χ*^2^-test.

b Log-rank test.

* According to Recist Criteria, CR=complete response; PR=partial response; SD=stable disease and PD=progressive disease.

**Table 3:** Univariate and multivariate Cox proportional hazard models for overall survival in mCRC. The landmark method determines each patient's HTN status at 3 months (HTN, *n*=48; no HTN, *n*=53). Survival time is calculated from that time point.

	**Univariate analysis**			**Multivariate analysis^a^**		
**Prognostic variable**	**HR**	**95% CI**	***P*-value**	**HR**	**95% CI**	***P*-value**
*No-HTN (*vs *HTN)*	0.58	0.38–0.88	0.011	0.53	0.34–0.84	0.007
*HTN at baseline*	0.86	0.53–1.40	0.537			
*WHO (*vs *2)*			(0.018)^a^			(0.188)
1	0.61	0.32–1.19	0.147	0.68	0.34–1.34	0.266
0	0.38	0.19–0.77	0.008	0.50	0.24–1.07	0.076
*Treatment line (*vs *3–7)*			(0.008)			(0.008)
2	0.65	0.39–1.08	0.093	0.78	0.45–1.38	0.397
1	0.41	0.24–0.72	0.002	0.39	0.21–0.74	0.004
*No. of metastatic sites (*vs *3–5)*			(0.013)			(0.012)
2	0.54	0.30–0.97	0.039	0.49	0.26–0.91	0.024
1	0.53	0.33–0.85	0.009	0.52	0.32–0.85	0.009
*Age (⩾60 years)*	0.87	0.57–1.31	0.497			
*Primary (*vs *colon) (rectal)*	0.99	0.65–1.50	0.950			
*Gender (*vs *male) (female)*	0.73	0.48–1.11	0.137			
*Type of chemotherapy (*vs *oxaliplatin)*			(0.002)			(0.268)
Irinotecan	0.87	0.46–1.64	0.663	0.83	0.42–1.67	0.608
5-Fluorouracil/bevacizumab	3.56	1.39–9.13	0.008	1.71	0.61–4.77	0.308
*Three weekly dosing schedule (*vs *two weekly)*	1.44	0.91–2.29	0.123			

Abbreviations: CI=confidence interval; HR=hazard ratio; HTN=hypertension; mCRC=metastatic colorectal cancer.

a Statistically significant (*P*=0.05) prognostic variables identified from the unvariate survival models were included in the multivariate model as covariates together with HTN. The *P*-value in parenthesis indicates the significance of a variable with more than two categories.

## References

[bib1] Bono P, Elfving H, Utriainen T, Osterlund P, Saarto T, Alanko T, Joensuu H (2009) Hypertension and clinical benefit of bevacizumab in the treatment of advanced renal cell carcinoma. Ann Oncol 20(2): 393–394 19211503 10.1093/annonc/mdn729

[bib2] Dahlberg SE, Sandler AB, Brahmer JR, Schiller JH, Johnson DH (2010) Clinical course of advanced non-small-cell lung cancer patients experiencing hypertension during treatment with bevacizumab in combination with carboplatin and paclitaxel on ECOG 4599. J Clin Oncol 28(6): 949–954 20085937 10.1200/JCO.2009.25.4482 PMC2834434

[bib3] Dvorak HF (2002) Vascular permeability factor/vascular endothelial growth factor: a critical cytokine in tumor angiogenesis and a potential target for diagnosis and therapy. J Clin Oncol 20(21): 4368–4380 12409337 10.1200/JCO.2002.10.088

[bib4] Hurwitz H, Fehrenbacher L, Novotny W, Cartwright T, Hainsworth J, Heim W, Berlin J, Baron A, Griffing S, Holmgren E, Ferrara N, Fyfe G, Rogers B, Ross R, Kabbinavar F (2004) Bevacizumab plus irinotecan, fluorouracil, and leucovorin for metastatic colorectal cancer. N Engl J Med 350(23): 2335–2342 15175435 10.1056/NEJMoa032691

[bib5] Ince WL, Jubb AM, Holden SN, Holmgren EB, Tobin P, Sridhar M, Hurwitz HI, Kabbinavar F, Novotny WF, Hillan KJ, Koeppen H (2005) Association of k-ras, b-raf, and p53 status with the treatment effect of bevacizumab. J Natl Cancer Inst 97(13): 981–989 15998951 10.1093/jnci/dji174

[bib6] Jubb AM, Oates AJ, Holden S, Koeppen H (2006) Predicting benefit from anti-angiogenic agents in malignancy. Nat Rev Cancer 6(8): 626–635 16837971 10.1038/nrc1946

[bib7] Maitland ML, Kasza KE, Karrison T, Moshier K, Sit L, Black HR, Undevia SD, Stadler WM, Elliott WJ, Ratain MJ (2009) Ambulatory monitoring detects sorafenib-induced blood pressure elevations on the first day of treatment. Clin Cancer Res 15(19): 6250–6257 19773379 10.1158/1078-0432.CCR-09-0058 PMC2756980

[bib8] Ranpura V, Pulipati B, Chu D, Zhu X, Wu S (2010) Increased risk of high-grade hypertension with bevacizumab in cancer patients: a meta-analysis. Am J Hypertens 23(5): 460–468 20186127 10.1038/ajh.2010.25

[bib9] Rini BI, Cohen DP, Lu D, Chen I, Hariharan S, Gore ME, Figlin RA, Baum MS, Motzer RJ (2010a) Hypertension (HTN) as a biomarker of efficacy in patients (pts) with metastatic renal cell carcinoma (mRCC) treated with sunitinib. Proc Am Soc Clin Oncol Genitourinary Cancer Symposium; (Abstract 312)

[bib10] Rini BI, Garcia JA, Cooney MM, Elson P, Tyler A, Beatty K, Bokar J, Ivy P, Chen HX, Dowlati A, Dreicer R (2010b) Toxicity of sunitinib plus bevacizumab in renal cell carcinoma. J Clin Oncol 28(17): e284–e285; author reply e286–e287 20439632 10.1200/JCO.2009.27.1759

[bib11] Scappaticci FA, Skillings JR, Holden SN, Gerber HP, Miller K, Kabbinavar F, Bergsland E, Ngai J, Holmgren E, Wang J, Hurwitz H (2007) Arterial thromboembolic events in patients with metastatic carcinoma treated with chemotherapy and bevacizumab. J Natl Cancer Inst 99(16): 1232–1239 17686822 10.1093/jnci/djm086

[bib12] Scartozzi M, Galizia E, Chiorrini S, Giampieri R, Berardi R, Pierantoni C, Cascinu S (2009) Arterial hypertension correlates with clinical outcome in colorectal cancer patients treated with first-line bevacizumab. Ann Oncol 20(2): 227–230 18842611 10.1093/annonc/mdn637

[bib13] Schneider BP, Wang M, Radovich M, Sledge GW, Badve S, Thor A, Flockhart DA, Hancock B, Davidson N, Gralow J, Dickler M, Perez EA, Cobleigh M, Shenkier T, Edgerton S, Miller KD (2008) Association of vascular endothelial growth factor and vascular endothelial growth factor receptor-2 genetic polymorphisms with outcome in a trial of paclitaxel compared with paclitaxel plus bevacizumab in advanced breast cancer: ECOG 2100. J Clin Oncol 26(28): 4672–4678 18824714 10.1200/JCO.2008.16.1612 PMC2653128

